# Comparison of Psoriatic Arthritis and Rheumatoid Arthritis Patients across Body Mass Index Categories in Switzerland

**DOI:** 10.3390/jcm10143194

**Published:** 2021-07-20

**Authors:** Enriqueta Vallejo-Yagüe, Theresa Burkard, Burkhard Möller, Axel Finckh, Andrea M. Burden

**Affiliations:** 1 Department of Chemistry and Applied Biosciences, Institute of Pharmaceutical Sciences, ETH Zurich, CH-8093 Zurich, Switzerland; enriqueta.vallejo@pharma.ethz.ch (E.V.-Y.); theresa.burkard@pharma.ethz.ch (T.B.); 2 Rheumatology, Immunology and Allergy, Inselspital, University Hospital of Bern, CH-3010 Bern, Switzerland; burkhard.moeller@insel.ch; 3 Division of Rheumatology, University Hospitals of Geneva, CH-1206 Geneva, Switzerland; axel.finckh@hcuge.ch

**Keywords:** psoriatic arthritis, rheumatoid arthritis, patient-reported outcomes, biologics

## Abstract

Abnormal body mass index (BMI) was associated with worse rheumatic markers in psoriatic arthritis (PsA) and rheumatoid arthritis (RA). Aiming to describe PsA and RA patients stratified by BMI, we performed a descriptive study in PsA and RA patients (two distinct cohorts) in the Swiss Clinical Quality Management in Rheumatic Diseases (SCQM) registry. New users of biologic or targeted synthetic disease-modifying anti-rheumatic drugs (b/tsDMARDs) were stratified by BMI at the start of their treatment (underweight, normal weight, overweight, obese). The PsA underweight and normal weight categories were merged. Age at disease onset and further characteristics at the start of the first b/tsDMARD treatment were compared across BMI categories vs. the corresponding normal weight group. The study included 819 PsA (36.5% overweight, 23.8% obese) and 3217 RA patients (4.4% underweight, 31.8% overweight, 17.0% obese). Compared to the corresponding normal weight group, PsA and RA obese patients had significantly (*p* < 0.05) higher C-reactive protein, worse disease activity, and lower quality of life (QoL). Obese PsA patients had significantly worse skin manifestation and pain, while obese RA patients had significantly higher erythrocyte sedimentation rate and tender joint counts, as well as lower seropositive prevalence. To conclude, obese PsA and RA patients presented worse disease activity and poorer QoL than those with normal weight.

## 1. Introduction

Obesity represents an increasing healthcare burden worldwide [1]; it affects approximately 15% of the European population [2] and 11% of the Swiss population [3]. Understanding obesity as a low-grade systemic inflammatory condition, where the white adipose tissue behaves as an endocrine organ secreting adipokines responsible for immune and inflammatory processes [4,5], suggests a common pathological pathway with immune-mediated inflammatory rheumatic diseases. 

Concerns about obesity or high body mass index (BMI) hindering the management of psoriatic arthritis (PsA) and rheumatoid arthritis (RA) patients have emerged in the past decade [4,6,7,8,9]. A higher prevalence of obesity was observed in PsA and RA patients compared to the general population [10,11,12,13], with PsA patients having the highest observed prevalence among both diseases [11,14]. Obesity was associated with worse disease activity and disease management in both PsA and RA [12,15,16,17,18,19,20,21] and a detrimental response to anti-tumor necrosis factor alpha (anti-TNF) treatments [15,17,18]. Additionally, despite the association of weight loss with a better disease outcome in PsA patients [22,23], a low BMI (underweight) was associated with worse RA disease activity [10,24]. 

Epidemiological studies using real-world data and clinical studies in PsA and RA patients rarely stratify by BMI, body weight, or body fat distribution. In a systematic review that included randomized clinical trials (RCTs) and observational studies assessing the failure to respond to anti-TNFs in adults with PsA, RA, spondyloarthropathies (SpA), and immune-mediated inflammatory diseases, the authors reported that less than 10% of eligible RCTs stratified by BMI at baseline [18]. 

We believe that there is interest and room for contribution to the scientific knowledge with regard to patient differences across BMI categories. Thus, we aimed to investigate differences in patient characteristics across BMI strata in patients with PsA and RA at the start of their first biologic or targeted synthetic disease-modifying anti-rheumatic drug (b/tsDMARD) treatment.

## 2. Materials and Methods

### 2.1. Study Design and Data Source

We performed a descriptive study of PsA and RA patients who were new users of b/tsDMARDs and were registered in the Swiss Clinical Quality Management in Rheumatic Diseases (SCQM) database. The SCQM registry [25], initiated in 1997, is a national longitudinal population-based cohort of rheumatic diseases in Switzerland that includes PsA and RA patients. Informed consent is obtained prior to enrolment and patients can withdraw their consent at any time. The data is generated during patient consultations and inserted by both the rheumatologist and the patient. The collected information includes physician- and patient-reported clinical endpoints (e.g., pain, skin manifestation, inflammatory markers), composite disease activity scores, health surveys, treatments, and comorbidities. Antirheumatic medication is recorded by the rheumatologist, including information on the start and stop dates of each treatment regimen. 

The study was reviewed by the ethics commission of the Canton of Zurich (KEK: Req-2020-00045). Since the researchers received pseudonymized data without access to the code key, a full ethics authorization was waived by the commission.

### 2.2. Study Population

PsA and RA patients registered in the SCQM database from 1 January 1997 to 31 July 2019 and starting their first b/tsDMARD treatment were included in the study. The first recorded start date of a b/tsDMARD treatment in the SCQM was defined as the index date. We excluded patients that started their b/tsDMARD treatment before the first registered visit in SCQM, as well as those without a measurement for weight and height at the index date (or within the 6 months prior to the index date). PsA and RA patients were treated as two distinct cohorts, which were analyzed separately but following a similar approach. 

### 2.3. Exposure

The primary exposure of interest in this analysis was patient BMI strata at the start of their first b/tsDMARD treatment. BMI (kg/m^2^) was calculated using the weight and height recorded at the index date, or as close as possible to this date within the previous 6 months (Supplementary Material Equation (S1)). We stratified patients by their BMI according to the World Health Organization (WHO) classification as follows: underweight (BMI < 18.5), normal weight (BMI 18.5–24.9), overweight (BMI 25.0–29.9), and obese (BMI ≥ 30) [26]. The exposures of interest were the abnormal BMI categories (underweight, overweight, and obese), and the normal weight group was set as the comparator group. 

### 2.4. Covariates

In both the PsA and RA patient cohorts, we included variables regarding disease onset, as well as patient characteristics, comorbidities, and medication use at the index date. Information on disease onset included age at first symptoms and age at diagnosis. Patient characteristics at the start of the first b/tsDMARD treatment (baseline characteristics) included demographics (e.g., sex, age, BMI, life habits), patient- and physician-reported clinical endpoints (e.g., pain, skin manifestation), composite disease activity scores, and health or quality of life (QoL) questionnaires. These variables were collected at the index date, or as close as possible to that date within a 6-month look-back window. Chronic comorbidities were included if they were ever reported in the patients’ records before or on the index date. Treatment with conventional synthetic disease-modifying anti-rheumatic drugs (csDMARDs) and glucocorticoids were identified if present on the index date. Disease-specific clinical endpoints and composite disease activity scores differed slightly between the PsA and RA patient cohorts. In both PsA and RA, we included the 28-joint disease activity score (DAS28) using the erythrocyte sedimentation rate (ESR) and DAS28 using C-reactive protein (CRP). While in the PsA cohort, minimal disease activity (MDA), the disease activity index for PsA (DAPSA), and the clinical DAPSA (cDAPSA) (DAPSA without CRP [27]) were additionally reported. Relevant formulas for composite disease activity scores and MDA are shown in the Supplementary Material Equations (S2)–(S6).

### 2.5. Data Analysis

All analyses were performed in each disease cohort separately. The BMI distribution was assessed in each disease cohort, and the prevalence of overweight and obese patients was plotted alongside the prevalence in the general Swiss population according to the Federal Statistical Office in Switzerland [3], stratifying by sex.

Patients’ age at disease onset and patient baseline characteristics were described in each disease cohort, stratifying by BMI category. Categorical variables were presented with counts (n—number of exposed patients) and percentages, and continuous variables were described using mean and standard deviation (SD) or median and interquartile range (IQR). Abnormal BMI categories were compared to the corresponding normal weight group using a chi-squared test for categorical variables and *t*-test or Wilcoxon test for continuous variables. For these tests, missing values did not function as a grouping variable, they were dropped instead. Statistical significance was defined as *p* < 0.05. 

Subsequently, among patients with a second b/tsDMARD treatment during the study period, we described the prescription patterns for the first and second b/tsDMARD treatments, stratifying by BMI. 

In a post hoc analysis, we assessed the duration of first b/tsDMARD treatment across BMI categories and the reasons for treatment discontinuation. In this analysis, only patients with an available treatment stop date (or a start date of a second and different b/tsDMARD) were included.

**Figure 1:**
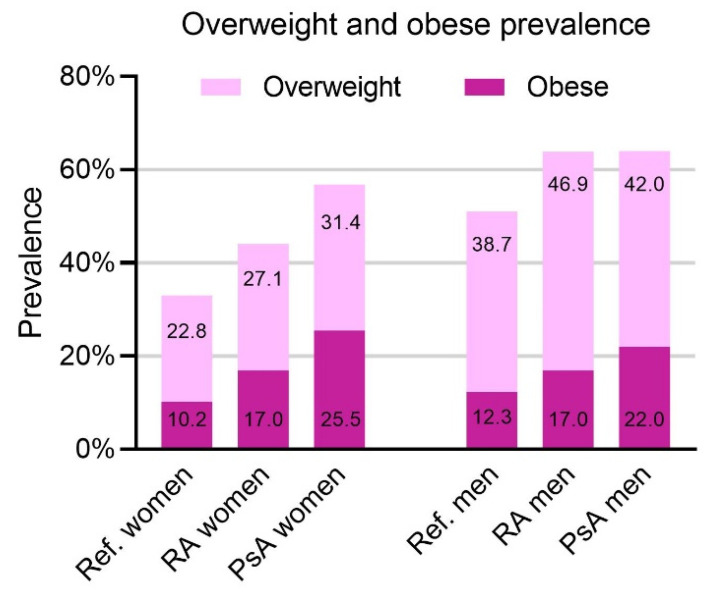
Overweight and obesity prevalence stratified by sex. The figure shows the findings from the studied rheumatoid arthritis (RA) and psoriatic arthritis (PsA) cohorts, along with the prevalence in the reference population (Ref.) according to the Swiss Federal Statistical Office, Übergewicht und Adipositas—Schweizerische Gesundheitsbefragung 2017—Korrigierte Version 25 September 2020|Publikation. Bundesamt für Statistik 2020. Available at: https://www.bfs.admin.ch/bfs/de/home/statistiken/gesundheit/erhebungen/sgb.assetdetail.14147705.html (accessed on 21 January 2021).

The analyses and figures were performed using R statistical software, R Foundation for Statistical Computing (Vienna, Austria) [28] version 3.5.2 (20 December 2018), except for Figure 1, which was plotted using GraphPad Prism version 9.0.2 for Windows, GraphPad Software (San Diego, CA USA) [29].

## 3. Results

We identified 4865 (1003 PsA and 3862 RA) patients in the SCQM between 1997 and 2019 who were new users of a b/tsDMARD and had available baseline information. From those, 829 patients were excluded due to missing weight and height information at the baseline. The remaining 819 PsA and 3217 RA patients were included in the study. A flow diagram reflecting the selection process is presented as Supplementary Figure S1. 

The prevalence of overweight and obese patients in our study, compared to the Swiss national population and stratified by sex, is shown in Figure 1. For both PsA and RA, the prevalence of obesity and overweight was higher than in the general Swiss population for both males and females. For PsA, obesity prevalence was 25.47% in women and 22.03% in men, and in RA, it was 17.0% in both women and men. 

### 3.1. Patient Characteristics

#### 3.1.1. Psoriatic Arthritis

Among the 819 PsA patients, only 13 patients (1.59%) had a BMI < 18.5; thus, due to the low numbers in the underweight category, we combined the underweight and normal weight groups in the PsA cohort. This resulted in 325 (39.68%) normal weight, 299 (36.51%) overweight, and 195 (23.81%) obese PsA patients (Table 1). Compared to the normal weight group, PsA patients categorized as overweight and obese were significantly older at the age of first symptoms and diagnosis.

Approximately half of the patients were women, with the lowest frequency of women in the underweight group (44.48%) and the highest in the normal weight category (56.31%). The mean age in the overweight group (50.55 years (SD 12.57)) was significantly higher than in the normal weight group (47.42 (SD 13.59)).

In the PsA cohort, anti-TNF drugs were the first b/tsDMARDs for 91.08% of normal weight, 91.64% of overweight, and 87.18% of obese patients (Table 2). Among those not treated with anti-TNFs, 68.97% normal weight, 64.00% overweight, and 68.00% obese patients received a tsDMARD (i.e., apremilast), and the remaining patients received a non-TNF biologic. History of cardiovascular event/disease and diabetes was more frequent in obese vs. the normal weight patients. Further information on the patients’ characteristics is provided in Supplementary Table S1. Here we show that the frequency of higher education and physical activity decreased with increasing BMI categories, but the frequency of smoking and alcohol consumption was similar between the BMI groups. 

Clinical characteristics of PsA patients at the start of the first b/tsDMARD treatment are presented in Table 3. Obese patients had significantly higher CRP than the normal weight category (*p* = 0.020), and no significant differences were observed in the rheumatoid factor (RF) and human leukocyte antigen B27 (HLA-B27) between the BMI strata. While the physician’s global disease activity assessment was similar between BMI strata, the physician´s global skin manifestation was significantly worse in the overweight and obese vs. the normal weight group (*p* < 0.02). Similarly, compared to the normal weight group, patient-reported disease activity (0 to 10) was significantly worse in the obese group, and both overweight and obese patients reported significantly worse joint pain (0 to 10). Conversely, the mean number of tender joint counts (TJC) was similar among BMI categories. Furthermore, the mean number of swollen joint counts (SJC) was significantly higher in the overweight vs. normal weight patients, but this was not consistent in the obese group. Additionally, no differences were observed across BMI groups regarding additional clinical manifestations (i.e., dactylitis, enthesitis, sacroilitis, spinal involvement, coxitis, peripheral arthritis, and nail manifestation).

The composite disease activity scores and health or QoL surveys of the PsA patients at the start of the first b/tsDMARD treatment are presented in Table 4. Overweight and obese patients presented worse disease activity, with significantly higher DAPSA (overweight 27.03 (SD 17.81), *p* = 0.022; obese 26.90 (SD 15.33), *p* = 0.037) compared to the normal weight group (23.23 (SD 15.46)). This was in line with the cDAPSA results and, likewise, significantly fewer patients had MDA in the overweight (1.67%, *p* = 0.002) and obese (2.05%, *p* = 0.026) vs. normal weight patients (6.77%). However, DAS28 was only significantly higher in the overweight group. Regarding body function and QoL, obese patients, in comparison to the normal weight group, had consistently significantly worse measures on the following surveys: Health Assessment Questionnaire (HAQ), European Quality of Life-5 dimensions (EuroQoL EQ-5D), Dermatology Life Quality Index (DLQI), and Short Form containing 12 items (SF12) physical component summary (pcs). However, this was not observed in the SF12 mental component summary (mcs). 

#### 3.1.2. Rheumatoid Arthritis

Among the 3217 RA patients, 142 (4.41%), 1505 (46.78%), 1024 (31.83%), and 546 (16.97%) patients were classified as underweight, normal weight, overweight, and obese, respectively. The age of the RA patients at disease onset is provided in Table 5. The patients categorized as overweight and obese were significantly older than the normal weight group at the age of first symptoms and diagnosis, and the underweight group was significantly younger at both dates.

Demographics, medication, and comorbidities of the RA patients at the start of their first b/tsDMARD treatment are described in Table 6. At the start of the first b/tsDMARD treatment, both overweight (mean 57.63 years (SD 12.28), *p* < 0.001) and obese patients (mean 57.04 years (SD 11.65), *p* < 0.001) were significantly older than the normal weight group (53.86 (SD 14.60)), and the underweight group was significantly younger (49.37 (SD 16.47), *p* = 0.001). The first b/tsDMARD was an anti-TNF in 89.44% of underweight, 87.51% of normal weight, 85.06% of overweight, and 84.07% of obese patients. Moreover, among those starting with another b/tsDMARD, 20.00% of underweight, 22.34% of normal weight, 24.18% of overweight, and 18.39% of obese patients received a tsDMARD, while the remaining patients received a non-TNF biologic. Prior history of cardiovascular event/disease, diabetes, other rheumatologic diseases, and depression were significantly more frequent in overweight and obese patients, and fractures were less frequent in obese patients. Other complementary information on patient characteristics is provided in Supplementary Table S2.

Clinical characteristics, composite disease activity scores, and health or QoL surveys of RA patients at the start of their b/tsDMARD treatment are presented in Table 7. Significantly fewer RF+ patients were observed in the obese (62.82%, *p* < 0.001) vs. normal weight groups (72.16%). Likewise, the prevalence of anti-CCP+ patients was significantly lower in obese (43.59%, *p* < 0.001) than in normal weight patients (48.64%). Obese patients presented significantly higher ESR and CRP levels than the normal weight group. While the physician´s global disease activity assessment remained similar across the RA BMI strata, the TJC were significantly increased in overweight and obese patients vs. the normal weight group, and this was not consistent with the SJC. 

Regarding disease activity, DAS28-ESR was significantly higher in overweight (4.39 (SD 1.41), *p* = 0.007) and obese (4.41 (SD 1.35), *p* = 0.011) in comparison to normal weight patients (4.23 (SD 1.42)), and this finding was in agreement with the DAS28-CRP results. Additionally, compared to normal weight patients, the overweight and obese patients presented worse QoL according to the HAQ, Euro-QoL, and SF12-pcs, but not SF12-mcs. Slightly worse HAQ and SF12-pcs were observed in the underweight patients; however, this was not statistically significant. 

### 3.2. Treatment Trends for the First and Second b/tsDMARD Treatments 

#### 3.2.1. Psoriatic Arthritis

**Figure 2:**
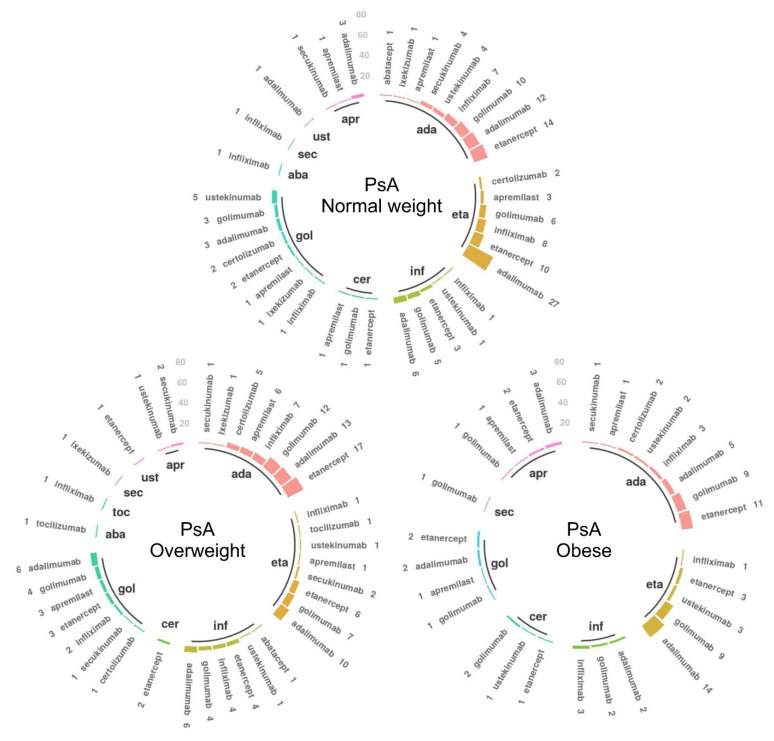
Treatment trends in the psoriatic arthritis (PsA) patients receiving a second biologic or targeted synthetic disease-modifying anti-rheumatic drug (b/tsDMARD), stratified by body mass index (*n* = 385). The inner circle illustrates the first b/tsDMARD, and for each corresponding initial drug, the bars indicate the second b/tsDMARD treatment. Only patients with recorded second treatments are represented. The number of patients for each drug sequence is mentioned by each bar. Complementary data are provided in Supplementary Table S3. Abbreviations: PsA—psoriatic arthritis; ada—adalimumab; eta—etanercept; inf—infliximab; cer—certolizumab; gol—golimumab; aba—abatacept; toc—tocilizumab; sec—secukinumab; ust—ustekinumab; apr—apremilast.

In the PsA cohort, 385 patients (47.00%) had a second b/tsDMARD following the stop of their first b/tsDMARD treatment. The distribution of paired first and second b/tsDMARDs used by these patients is illustrated in Figure 2, and counts to complement the figure are provided in the Supplementary Table S3. Among those with a recorded second b/tsDMARD treatment, 94.84% of normal weight, 95% of overweight, and 90% of obese patients were treated with an anti-TNF as their first b/tsDMARD. Among the patients starting with an anti-TNF treatment, 84.35% of normal weight, 85.71% of overweight, and 88.89% of obese patients moved to the same or another anti-TNF as their second treatment. The most common second b/tsDMARDs across the four BMI categories continued being the anti-TNFs adalimumab, etanercept, golimumab, and infliximab. 

In the post hoc analysis, we identified 451 patients (175 normal weight patients, 173 overweight, and 103 obese) with an available stopping date for their first b/tsDMARD treatment. Among these, the median years of treatment were 11.66 (IQR (3.91, 24.28)), 11.47 (IQR (5.22, 26.68)), and 9.59 (IQR (3.94, 20.78)) for normal weight, overweight, and obese PsA patients, respectively. No statistically significant differences were found in the duration of treatment across BMI groups. The reasons for stopping the first b/tsDMARD treatment (Supplementary Table S1) were statistically different in the obese (*p* = 0.024) versus normal weight strata.

#### 3.2.2. Rheumatoid Arthritis

**Figure 3:**
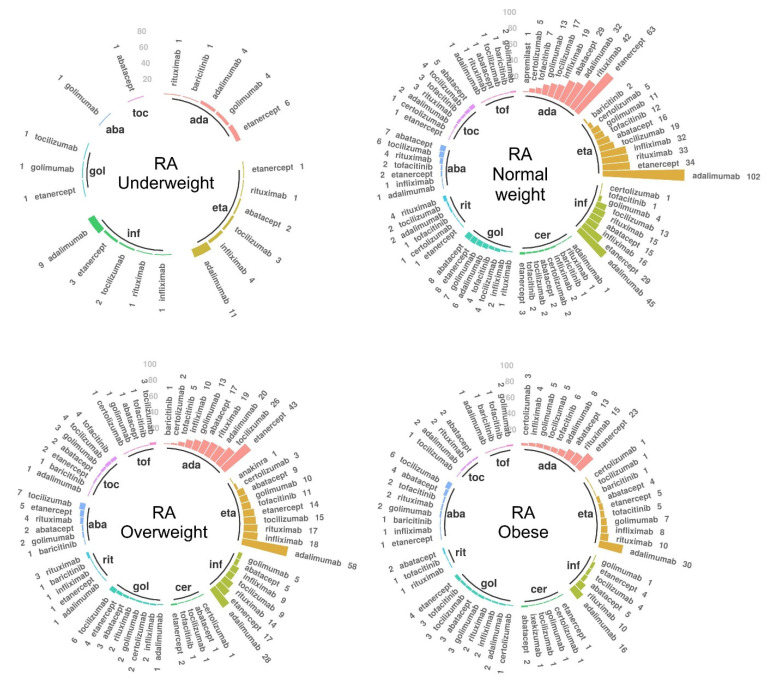
Treatment trends in the rheumatoid arthritis (RA) patients receiving a second biologic or targeted synthetic disease-modifying anti-rheumatic drug (b/tsDMARD), stratified by body mass index (*n* = 1546). The inner circle illustrates the first b/tsDMARD, and for each corresponding initial drug, the bars indicate the second b/tsDMARD treatment. Only patients with recorded second treatments are represented. The number of patients for each drug sequence is mentioned by each bar. Complementary data are provided in Supplementary Table S4. Abbreviations: ada—adalimumab; eta—etanercept; inf—infliximab; cer—certolizumab; gol—golimumab; rit—rituximab; aba—abatacept; toc—tocilizumab; tof—tofacitinib.

In the RA cohort, 1546 patients (48.06%) received a second b/tsDMARD following the stop of their first b/tsDMARD treatment. Their distribution of paired first and second treatments are shown in Figure 3, complemented with numerical values in Supplementary Table S4. Following anti-TNFs as first treatment (as was the case in 96.61% of underweight patients, 91.99% of normal weight, 89.14% of overweight, 86.49% of obese), a total of 78.95% of underweight, 64.15% of normal weight, 61.83% of overweight, and 58.04% of obese patients continued with anti-TNF as a second b/tsDMARD. Overall, the most commonly used treatments as a second b/tsDMARD were adalimumab and etanercept, except for the obese group, where rituximab was more frequently used than etanercept. 

The post hoc analysis identified 1787 patients (856 normal weight, 557 overweight, 307 obese, and 67 underweight) with available stop dates for their first b/tsDMARD treatment. Among these, the median years of treatment was 13.65 (IQR (5.79, 29.93)), 13.24 (IQR (6.01, 32.03)), 11.70 (IQR (5.31, 22.83)), and 12.19 (IQR (5.14, 35.09)) years for patients with normal weight, overweight, obesity, and underweight, respectively. In comparison to the normal weight group, obese patients had a significantly shorter duration of treatment (*p* = 0.006). The distribution of reasons for stopping the first b/tsDMARD treatment (Supplementary Table S2) was significantly different in obese vs. normal weight patients (*p* = 0.007).

## 4. Discussion

To our knowledge, this is one of the largest studies to examine differences in patient characteristics between PsA patients across BMI strata, and we additionally provided a comparison to the RA population. In our analysis, we identified that obese patients were generally older at disease onset and they had significantly higher CRP levels, worse disease activity scores, and lower QoL at the time of starting their first b/tsDMARD treatment compared to normal weight patients. Obese PsA patients also had worse skin manifestations and reported higher pain compared to the normal weight group. Meanwhile, in the RA cohort, the obese patients had higher ESR, higher TJC, but similar SJC, and smaller prevalence of RF+ patients. In both cohorts, anti-TNF drugs were the most commonly prescribed b/tsDMARDs across every BMI category, and >84% PsA and >58% RA patients moved to the same or another anti-TNF (assuming gaps of ≥1 month as treatment stoppages). 

### 4.1. Prevalence of BMI Strata

We identified a higher obesity prevalence in our PsA (23.8% obese) and RA (17.0% obese) patient cohorts in comparison to the general Swiss population (Switzerland 2017, 11% obesity) [3]. This is in accordance with prior studies. For example, obesity prevalence was 32% among PsA patients from Danish and Icelandic registries [15] compared to the 14–17% and 22% of the general Danish and Icelandic populations, respectively [15,30,31]. In Canadian studies, obesity prevalence was 35.4–37% in PsA [12,13] and 28% in RA vs. 18% in the general population [11]. Furthermore, among RA patients in German cohorts, obesity prevalence was 21.4–23.8% vs. the 18.2% in the general population [10]. While we observed a lower prevalence of obesity in both our cohorts compared to the above-mentioned studies, this may be explained by the less frequent obesity in our reference population. Additionally, our findings confirmed the higher obesity prevalence among PsA vs. RA patients, which were previously observed by other studies [11,14].

While we aimed to provide stratified information on underweight patients in both cohorts, we were unable to do so for the PsA cohort due to the small sample size. With 13 patients being underweight (BMI < 18.5), the prevalence in our cohort was 1.59%. Due to the lack of existing information on underweight PsA patients, it is unclear whether this is comparable across international patient cohorts. Conversely, in our RA cohort, 4.4% of patients were categorized as underweight. Comparing this prevalence with that in other studies, we observed a wide range of findings. In German cohorts, the prevalence of underweight patients was 1.1–2.2% in RA vs. 0.8% in the reference population [10]. In a Dutch cohort of active RA, 8 patients (9.0%) had BMI < 20 kg/m^2^ [32], and in a study in the United States, 38 patients (4.9%) had BMI < 20 kg/m^2^ [33]. While there seems to be a lack of agreement on the prevalence of underweight patients in RA, this may be explained by the use of different BMI thresholds and measuring time-points throughout the course of the disease, the potentially different underweight prevalence in the reference populations, and the restrictions posed by the study-specific inclusion/exclusion criteria. Further evidence on underweight patients in both PsA and RA are certainly warranted.

### 4.2. Patient Characteristics

Previous studies reported that overweight and obese PsA and RA patients were older than the normal weight patients at disease onset [10,13], and this was similarly observed in our study. While the reasons for a later onset age are unclear, it could be the consequence of failing to recognize rheumatic clinical signs when they could be attributed to heavy body weight [13]. 

In an observational cohort study in Danish and Icelandic registries including 1271 PsA patients starting anti-TNFs, the authors reported that obese patients were older and had higher CRP levels, TJC, DAS28, HAQ, pain levels, and global disease assessments than non-obese patients at the baseline, but not significantly higher SJC [15]. Similarly, in our study, obese PsA patients had higher CRP levels, worse pain, and worse HAQ when compared to the normal weight group. However, SJC and DAS28 were only significantly higher in the overweight group, despite DAPSA showing significantly worse disease activity in both overweight and obese patients. This inconsistency between the PsA-specific DAPSA and the RA-derived DAS28 without specific adaptations to PsA in the obese group may be explained by the different components contributing to each score. First, DAPSA includes patient assessments of disease activity and pain, which were significantly higher in obese patients. Second, if the potential underestimation of SJC in obese patients due to excess fat tissue around the joints has a higher impact on the resulting DAS28 score than in DAPSA, this may also contribute to the significantly worse observed DAPSA score in both overweight and obese patients, but only significantly worse DAS28 in overweight compared to the normal weight patients. 

Among the RA patients, some studies distinguished subjective (e.g., TJC, patient global assessment, and pain) and objective (e.g., CRP level, ESR, and SJC) endpoints when comparing obese and non-obese patients [19,34]. For example, in a systematic review and meta-analysis in which DAS28 and HAQ were higher in obese patients, the authors suggested that the increased disease activity was mainly due to elevated subjective score components, such as TJC, and global pain and health assessments [34]. Additionally, another systematic review and meta-analysis also suggested that obesity may influence patient global disease assessment, pain, and QoL, but agreed that SJC are not higher in obese RA patients [16]. Albrecht et al. similarly observed increased DAS28 but not SJC among obese RA patients in comparison to the normal weight ones, but conversely, with other studies, they also found higher ESR in obese patients [10]. In line with this, we identified worse DAS28, lower QoL, higher TJC, and no differences in SJC in obese compared to normal weight RA patients, but we also identified increased CRP levels and ESR in the obese vs. normal weight patients. Thus, we believe that the observed increased DAS28 in obese patients with RA was not only due to TJC, previously described as a subjective endpoint, but also due to the enhanced inflammatory markers (CRP level and ESR), or so-called objective end-points.

The discussion of objective and subjective endpoints is likely important for the assessment of obese rheumatology patients. While in the clinic, SJC is considered an objective measure, the excess or absence of fat tissue around the joints may influence the assessment of swelling, adding complexity to the practice and potentially reducing the reliability of this measure in obese patients. This may explain the inconsistency observed between SJC and TJC among RA patients, as well as the controversial higher SJC in overweight vs. obese among PsA patients. Additionally, in our RA cohort, we observed a tendency of higher SJC and lower TJC in underweight compared to normal weight patients, which contrasts the findings in obese patients. This SJC trend in underweight RA patients was also observed in German cohorts, along with heterogeneous results for TJC [10]. Following the above-discussed potential impact of fat mass in the SJC, we believe that higher SJC in underweight patients could be the consequence of a more easily detectable inflammation during clinical assessment and, conversely, SJC could potentially be underestimated in obese patients. Conversely, one may also consider that the disagreement between higher TJC, but similar SJC, in obese RA patients could be alternatively explained by hyperalgesia due to mood disorders, such as depression, and subsequent fibromyalgia, which has been associated with obesity [35].

Overall, obesity was associated with pain, lower QoL, higher disability, depression [36,37], cardiovascular risk [38], and diabetes [39] in the general population. Obesity (adiposity) was identified as a risk factor for PsA [40] and RA [41], and a higher frequency of lipid abnormalities (higher dyslipidemia and serum triglycerides and lower high-density lipoprotein (HDL)-cholesterol) was identified in PsA patients [42]. This, together with the evidence of white adipose tissue enhancing immune and inflammatory processes [4,5,43], and with the higher prevalence of obesity in PsA and RA patients vs. the general population [10,11,12,13], support the reason to believe that both the obesity and the rheumatology disease contribute to the patient status, and therefore may play a role in the assessed worse QoL and fragility observed in these patients. Additionally, the disagreement on the findings through QoL surveys, including physical components (SF12-pcs, Euro-QoL), vs. the SF12-mcs, which solely assesses mental components, suggests that the findings of worse QoL in PsA/RA obese patients may be driven by the physical restrictions and less so from potentially decreased mental wellbeing. 

### 4.3. Treatment Trends

Existing studies have shown that obesity may be associated with a detrimental response to anti-TNF treatments in PsA and RA patients in comparison to non-obese or normal weight patients [15,17,18]. Conversely, it has been suggested that high BMI does not influence the response to abatacept [44,45,46,47], rituximab [48], and tocilizumab [49,50] in RA. Thus, while we observed that anti-TNFs were the most frequent choice of b/tsDMARD treatment across every BMI category in both cohorts, there was a slight tendency for lower anti-TNF use among RA patients in higher BMI categories, which could indicate that clinical decision-making is in line with these previous findings. However, this cannot be confirmed based on our results, and a time-series analysis may be more appropriate. 

### 4.4. Strengths and Limitations

This is one of the largest studies that assessed differences in PsA patients across BMI categories and provides a further comparison with RA patients. To the best of our knowledge, the SCQM database is one of the few rheumatic disease registries with relatively complete information on patient weight and height, thereby enabling a stratification by BMI categories. Additionally, with obesity rates differing between countries [3,51], we consider the Swiss population a sample of interest due to its relatively low, although increasing, prevalence of obesity in the general population (2017, 11%) [3]. However, BMI does not provide information on body composition [52], and therefore, we acknowledge the potential misclassification for those patients for whom the BMI categories do not fairly represent their fat distribution. Ideally, we would like to have data from the hip/waist circumference, the skinfold thickness, and the bioelectrical impedance, which would provide a better assessment of unhealthy weight. However, this was not available in our data. Moreover, this information is rarely recorded during clinical practice; thus, this is likely unrealistic to be present in real-world data. Missing information regarding body mass composition may be especially relevant for the underweight group, resulting in a lack of figures on cachexia (abnormal body composition). In cachexic patients, body fat (particularly belly fat) may remain stable or even increased despite muscle loss and weight loss [53]. Thus, it is possible that systemic inflammation in cachexic underweight patients may overlap with the rheumatic inflammation in a similar manner than in obese patients, despite the different phenotype. This could be better captured by more precise measures of body fat composition. However, we did not observe aggravated inflammatory markers in the underweight category vs. the normal weight patients, which may suggest the absence of additional systemic inflammation in these patients. 

While the SCQM is a comprehensive dataset, we acknowledge that there are limitations regarding data completeness. We may have incomplete information on non-rheumatic comorbidities, as this information is self-reported by the patient and reporting systems have changed since the launch of the SCQM. For example, while previous evidence suggested that the prevalence of depression was 9–22% in PsA [54,55,56] and 14–38% in RA patients [57], we observed a lower prevalence (4–5.7% in PsA and 3.5–7.5% in RA), supporting the belief that comorbidities may be underreported in our cohorts.

In both PsA and RA patients, we observed that obese patients were generally older than the normal weight patients, which could be due to their increasing BMI with age, and therefore the interplay between age, BMI, and disease activity in PsA and RA patients deserves further attention. Additionally, we stratified patients according to their BMI at the start of their first b/tsDMARD treatment; thus, for our analysis of differences in age at disease onset, we assumed that BMI remained constant from disease onset to the start of their b/tsDMARD treatment. 

Finally, the decision regarding what qualifies as a true treatment stop, or which length of treatment-free gap (between stop and restart of the same drug agent) should be considered as treatment continuation or as a true stop and restart may be arguable. In this study, we accepted a one-month grace period, whereby patients with a stop and restart of the same b/tsDMARD agent were considered as having continuous use. Therefore, the observed high proportion of patients with the same first and second b/tsDMARDs may be explained by a misclassified stop (e.g., drug holidays), but it may as well be indicative of patients restarting a therapy they previously did well on. Further research on the patterns of stopping b/tsDMARDs would be of interest.

## 5. Conclusions

In conclusion, this study provided a clinical picture of PsA and RA patients in Switzerland across different BMI categories. Obesity prevalence was higher in PsA and RA compared to the general Swiss population, and PsA and RA patients with obesity had worse disease activity and lower QoL in comparison with the corresponding normal weight groups. In the PsA cohort, the findings on disease activity (DAPSA) in obese patients were mainly driven by CRP levels and the patient assessment on disease activity and pain, but the results remained consistent when excluding CRP levels from the equation (cDAPSA). In the RA cohort, the results on disease activity (DAS28) in obese patients were primarily attributable to TJC and ESR or CRP levels. Finally, these findings suggest that BMI should be considered when treating or studying PsA and RA patients. 

## Figures and Tables

**Table 1:** Age at disease onset in the psoriatic arthritis (PsA) patient cohort.

	Normal Weight	Overweight		Obese	
	(*n* = 325)	(*n* = 299)	*p*	(*n* = 195)	*p*
Age at first symptoms (mean (SD))	38.04 (14.33)	41.35 (13.75)	0.004	40.73 (12.17)	0.031
Missing	7 (2.15)	8 (2.68)		4 (2.05)	
Age at diagnosis (mean (SD))	41.81 (14.39)	45.21 (13.09)	0.002	45.00 (11.58)	0.010
Missing	5 (1.54)	5 (1.67)		5 (2.56)	

Values are the number and column percentages, unless otherwise specified. The underweight, overweight, and obese categories were compared to the normal weight group using *t*-test. For the test, the missing values did not function as a grouping variable. Abbreviations: *n*—sample size; SD—standard deviation.

**Table 2:** Demographics, medication, and comorbidities of the psoriatic arthritis (PsA) patients at the start of their first biologic or targeted synthetic disease-modifying anti-rheumatic drug (b/tsDMARD) treatment.

	Normal Weight	Overweight		Obese	
	(*n* = 325)	(*n* = 299)	*p*	(*n* = 195)	*p*
Women	183 (56.31)	133 (44.48)	0.004	108 (55.38)	0.909
Age, years (mean (SD))	47.42 (13.59)	50.55 (12.57)	0.003	49.47 (10.80)	0.073
PsA duration, years (median [IQR])	2.47 [0.60, 7.05]	2.67 [0.66, 7.58]	0.969	1.79 [0.68, 5.80]	0.290
Missing	5 (1.6)	5 (1.67)		5 (2.56)	
First b/tsDMARD			0.900		0.369
Anti-TNF biologic	296 (91.08)	274 (91.64)		170 (87.18)	
Other biologic	9 (2.77)	9 (3.01)		8 (4.1)	
tsDMARD	20 (6.15)	16 (5.35)		17 (8.72)	
csDMARD on index date	157 (48.31)	153 (51.17)	0.526	105 (53.85)	0.258
Glucocorticoids on index date	40 (12.31)	38 (12.71)	0.976	18 (9.23)	0.350
Other rheumatological disease	32 (9.85)	37 (12.37)	0.380	26 (13.33)	0.281
Fractures, surgeries	6 (1.85)	2 (0.67)	0.342	1 (0.51)	0.377
Skin problems, allergies, drug reactions	36 (11.08)	47 (15.72)	0.112	25 (12.82)	0.647
Infections	4 (1.23)	7 (2.34)	0.454	2 (1.03)	1.000
Cancerous tumor	7 (2.15)	5 (1.67)	0.884	7 (3.59)	0.484
Cardiovascular event/disease	27 (8.31)	40 (13.38)	0.056	33 (16.92)	0.005
Diabetes	4 (1.23)	8 (2.68)	0.307	10 (5.13)	0.017
Other metabolic problems	6 (1.85)	13 (4.35)	0.113	7 (3.59)	0.346
Depression/anxiety	13 (4)	17 (5.69)	0.426	10 (5.13)	0.700

Values are the number and column percentages, unless otherwise specified. Significance tests compared the overweight and obese categories to the normal weight group using chi-squared test for categorical variables and *t*-test for continuous variables, except for the Wilcoxon test for the PsA duration. For these tests, the missing values did not function as a grouping variable. Abbreviations: *n*—sample size; SD—standard deviation; IQR—interquartile range; anti-TNF—anti-tumor necrosis factor; tsDMARD—targeted synthetic disease-modifying anti-rheumatic drug; csDMARD—conventional synthetic disease-modifying anti-rheumatic drug. Note: csDMARD and glucocorticoids indicate use on the index date, and not as ever having used them before.

**Table 3:** Clinical characteristics of psoriatic arthritis (PsA) patients at the start of their first biologic or targeted synthetic disease-modifying anti-rheumatic drug (b/tsDMARD) treatment.

	Normal Weight	Overweight		Obese	
	(*n* = 325)	(*n* = 299)	*p*	(*n* = 195)	*p*
RF+	14 (4.31)	10 (3.34)	0.667	5 (2.56)	0.423
Missing	88 (27.08)	80 (26.76)		52 (26.67)	
HLA-B27+	40 (12.31)	28 (9.36)	0.319	22 (11.28)	1.000
Missing	150 (46.15)	142 (47.49)		98 (50.26)	
ESR mm/h (median [IQR])	10 (5, 21.75)	12 (6., 22.25)	0.104	14.5 (6, 23)	0.081
Missing	31 (9.54)	27 (9.03)		13 (6.67)	
CRP mg/dL (median [IQR])	0.5 (0.2, 0.9)	0.60 (0.3, 1.10)	0.152	0.79 (0.40, 1.20)	0.020
Missing	40 (12.31)	36 (12.04)		18 (9.23)	
Physician global disease activity (mean (SD))	4.43 (2.03)	4.56 (1.86)	0.414	4.43 (1.85)	0.991
Missing	17 (5.23)	9 (3.01)		6 (3.08)	
Physician global skin manifestation (0–3) (mean (SD))	0.93 (0.85)	1.11 (0.84)	0.012	1.12 (0.85)	0.019
Missing	30 (9.23)	17 (5.69)		14 (7.18)	
Patient-reported disease activity (0–10) (mean (SD))	5.13 (2.72)	5.46 (2.54)	0.178	5.97 (2.60)	0.003
Missing	89 (27.38)	59 (19.73)		48 (24.62)	
Joint pain last 24 h (0–10) (mean (SD))	4.90 (2.65)	5.41 (2.39)	0.028	6.11 (2.41)	<0.001
Missing	82 (25.23)	56 (18.73)		46 (23.59)	
Number tender joints 28 (0–28) (mean (SD))	4.21 (5.11)	4.90 (5.52)	0.109	4.35 (5.10)	0.767
Missing	12 (3.69)	6 (2.01)		10 (5.13)	
Number tender joints 68 (0–68) (mean (SD))	8.07 (9.08)	8.97 (10.23)	0.267	8.65 (9.63)	0.518
Missing	41 (12.62)	20 (6.69)		21 (10.77)	
Number swollen joints 28 (0–28) (mean (SD))	2.73 (3.39)	3.65 (4.57)	0.005	2.88 (3.36)	0.627
Missing	8 (2.46)	6 (2.01)		8 (4.1)	
Number swollen joints 66 (0–66) (mean (SD))	4.62 (5.22)	5.78 (7.1)	0.026	4.76 (5.30)	0.769
Missing	41 (12.62)	21 (7.02)		21 (10.77)	
Musculoskeletal manifestations	269 (82.77)	242 (80.94)	0.624	163 (83.59)	0.904
Manifestation: dactylitis	114 (35.08)	119 (39.8)	0.256	80 (41.03)	0.206
Manifestation: enthesitis	138 (42.46)	120 (40.13)	0.611	81 (41.54)	0.909
Manifestation: sacroiliitis	80 (24.62)	71 (23.75)	0.873	34 (17.44)	0.071
Manifestation: spinal involvement	91 (28)	78 (26.09)	0.655	48 (24.62)	0.458
Manifestation: coxitis	18 (5.54)	9 (3.01)	0.176	17 (8.72)	0.222
Manifestation: peripheral arthritis	170 (52.31)	158 (52.84)	0.957	113 (57.95)	0.246
Nail manifestation	68 (20.92)	71 (23.75)	0.453	56 (28.72)	0.056

Values are the number and column percentages, unless otherwise specified. Significance tests compared the overweight and obese categories to the normal weight group using chi-squared test for categorical variables and *t*-test for continuous variables, except for the Wilcoxon test for ESR and CRP. For the tests, the missing values did not function as a grouping variable. Abbreviations: *n*—sample size; SD—standard deviation; IQR—interquartile range; RF+—rheumatoid factor positive; HLA-B27+—human leukocyte antigen B27 positive; ESR—erythrocyte sedimentation rate; mm/h—millimeters per hour; CRP—C-reactive protein; mg/dL—milligrams per deciliter.

**Table 4:** Composite disease activity scores and health or quality of life surveys in psoriatic arthritis (PsA) patients at the start of their first biologic or targeted synthetic disease-modifying anti-rheumatic drug (b/tsDMARD) treatment.

	Normal Weight	Overweight		Obese	
	(*n* = 325)	(*n* = 299)	*p*	(*n* = 195)	*p*
MDA	22 (6.77)	5 (1.67)	0.002	4 (2.05)	0.026
Missing	64 (19.69)	41 (13.71)		36 (18.46)	
DAPSA (mean (SD))	23.23 (15.46)	27.03 (17.81)	0.022	26.90 (15.33)	0.037
Missing	118 (36.31)	95 (31.77)		72 (36.92)	
cDAPSA without CRP (mean (SD))	22.16 (14.95)	25.64 (17.21)	0.023	26.03 (14.89)	0.020
Missing	106 (32.62)	72 (24.08)		65 (33.33)	
DAS28-ESR (mean (SD))	3.30 (1.27)	3.57 (1.32)	0.014	3.43 (1.23)	0.273
Missing	44 (13.54)	34 (11.37)		24 (12.31)	
DAS28-CRP (mean (SD))	3.26 (1.12)	3.52 (1.18)	0.011	3.41 (1.09)	0.191
Missing	51 (15.69)	42 (14.05)		24 (12.31)	
HAQ (mean (SD))	0.71 (0.65)	0.75 (0.58)	0.375	0.89 (0.61)	0.003
Missing	53 (16.31)	46 (15.38)		38 (19.49)	
Euro-QoL (mean (SD))	65.32 (17.81)	63.51 (17.38)	0.366	60.33 (20.31)	0.037
Missing	169 (52.00)	145 (48.49)		90 (46.15)	
DLQI (mean (SD))	3.53 (5.35)	4.62 (6.08)	0.087	5.52 (7.66)	0.013
Missing	167 (51.38)	137 (45.82)		87 (44.62)	
SF12-pcs (mean (SD))	39.06 (10.54)	38.23 (9.94)	0.368	35.78 (9.23)	0.001
Missing	67 (20.62)	64 (21.4)		41 (21.03)	
SF12-mcs (mean (SD))	45.96 (11.36)	45.48 (11.46)	0.640	44.12 (11.67)	0.116
Missing	67 (20.62)	64 (21.4)		41 (21.03)	

Values are the number and column percentages, unless otherwise specified. Significance tests compared the overweight and obese categories to the normal weight group using chi-squared test for categorical variables and *t*-test for continuous variables. For the tests, the missing values did not function as a grouping variable. Abbreviations: *n*—sample size; SD—standard deviation; MDA—minimal disease activity; DAPSA—Disease Activity Index for Psoriatic Arthritis; DAS28—28-joint Disease Activity Score; ESR—erythrocyte sedimentation rate; CRP—C-reactive protein; HAQ—Health Assessment Questionnaire; Euro-QoL—European Quality of Life instrument; DLQI—Dermatology Life Quality Index; SF12—Short-Form 12 health survey; pcs—physical component summary; mcs—mental component summary.

**Table 5:** Age at disease onset in the rheumatoid arthritis (RA) patient cohort.

	Normal Weight	Overweight		Obese		Underweight	
	(*n* = 1505)	(*n* = 1024)	*p*	(*n* = 546)	*p*	(*n* = 142)	*p*
Age at first symptoms, years (mean (SD))	43.90 (15.46)	48.42 (13.98)	<0.001	48.52 (12.59)	<0.001	37.78 (16.68)	<0.001
Missing	34 (2.26)	39 (3.81)		21 (3.85)		7 (4.93)	
Age at diagnosis, years (mean (SD))	45.36 (15.31)	50.10 (13.71)	<0.001	49.95 (12.67)	<0.001	39.86 (16.87)	<0.001
Missing	34 (2.26)	33 (3.22)		19 (3.48)		8 (5.63)	

Values are the number and column percentages, unless otherwise specified. The underweight, overweight, and obese categories were compared to the normal weight group using *t*-test. For the test, the missing values did not function as a grouping variable. Abbreviations: *n*—sample size; SD—standard deviation.

**Table 6:** Demographics, medication, and comorbidities of the rheumatoid arthritis (RA) patients at the start of their first biologic or targeted synthetic disease-modifying anti-rheumatic drug (b/tsDMARD) treatment.

	Normal Weight	Overweight		Obese		Underweight	
	(*n* = 1505)	(*n* = 1024)	*p*	(*n* = 546)	*p*	(*n* = 142)	*p*
Women	1237 (82.19)	664 (64.84)	<0.001	416 (76.19)	0.003	133 (93.66)	0.001
Age, years (mean (SD))	53.86 (14.60)	57.63 (12.28)	<0.001	57.04 (11.65)	<0.001	49.37 (16.47)	0.001
RA duration, years (median [IQR])	4.82(1.67, 12.32)	4.08(1.35, 10.75)	0.005	3.73(1.38, 8.77)	0.001	6.94(2.16, 14.47)	0.115
Missing	34 (2.26)	33 (3.22)		19 (3.48)		8 (5.63)	
First b/tsDMARD			0.192		0.095		0.785
Anti-TNF biologic	1317 (87.51)	871 (85.06)		459 (84.07)		127 (89.44)	
Other biologic	146 (9.7)	116 (11.33)		71 (13.00)		12 (8.45)	
tsDMARD	42 (2.79)	37 (3.61)		16 (2.93)		3 (2.11)	
csDMARD on index date	1000 (66.45)	704 (68.75)	0.242	394 (72.16)	0.016	92 (64.79)	0.759
Glucocorticoids on index date	604 (40.13)	427 (41.7)	0.456	222 (40.66)	0.870	49 (34.51)	0.222
Other rheumatological disease	308 (20.47)	268 (26.17)	0.001	156 (28.57)	<0.001	22 (15.49)	0.192
Fractures, surgeries	151 (10.03)	80 (7.81)	0.067	36 (6.59)	0.021	14 (9.86)	1.000
Skin problems, allergies, drug reactions	18 (1.2)	11 (1.07)	0.927	8 (1.47)	0.796	2 (1.41)	1.000
Infections	22 (1.46)	17 (1.66)	0.816	12 (2.2)	0.338	1 (0.7)	0.718
Cancerous tumor	27 (1.79)	26 (2.54)	0.253	12 (2.2)	0.683	1 (0.7)	0.535
Cardiovascular event/disease	216 (14.35)	274 (26.76)	<0.001	185 (33.88)	<0.001	13 (9.15)	0.113
Diabetes	36 (2.39)	49 (4.79)	0.002	48 (8.79)	<0.001	2 (1.41)	0.650
Other metabolic problems	36 (2.39)	55 (5.37)	<0.001	33 (6.04)	<0.001	0 (0)	0.118
Depression/anxiety	58 (3.85)	58 (5.66)	0.041	41 (7.51)	0.001	5 (3.52)	1.000

Values are the number and column percentages, unless otherwise specified. Significance tests compared the underweight, overweight, and obese categories to the normal weight group using chi-squared test for categorical variables and *t*-test for continuous variables, except for the Wilcoxon test for the RA duration. For the tests, the missing values did not function as a grouping variable. Abbreviations: *n*—sample size; SD—standard deviation; IQR—interquartile range; anti-TNF—anti-tumor necrosis factor; tsDMARD—targeted synthetic disease-modifying anti-rheumatic drug; csDMARD—conventional synthetic disease-modifying anti-rheumatic drug. Note: csDMARD and glucocorticoids indicate use on the index date, and not as ever having used them before.

**Table 7:** Clinical characteristics, composite disease activity scores, and health or quality of life surveys of rheumatoid arthritis (RA) patients at the start of their first biologic or targeted synthetic disease-modifying anti-rheumatic drug (b/tsDMARD) treatment.

	Normal Weight	Overweight		Obese		Underweight	
	(*n* = 1505)	(*n* = 1024)	*p*	(*n* = 546)	*p*	(*n* = 142)	*p*
RF+	1086 (72.16)	700 (68.36)	0.219	343 (62.82)	<0.001	101 (71.13)	0.755
Missing	53 (3.52)	58 (5.66)		28 (5.13)		4 (2.82)	
Anti-CCP+	732 (48.64)	489 (47.75)	0.317	238 (43.59)	<0.001	61 (42.96)	0.732
Missing	427 (28.37)	278 (27.15)		136 (24.91)		49 (34.51)	
ESR mm/h (median [IQR])	18 (9, 32)	20 (10, 34)	0.103	20(10, 33)	0.026	18 (8, 38.75)	0.919
Missing	72 (4.78)	37 (3.61)		27 (4.95)		4 (2.82)	
CRP mg/dL (median [IQR])	0.8(0.30, 1.4)	0.8(0.3, 1.6)	0.089	0.9(0.4, 1.52)	0.005	0.8(0.2, 1.50)	0.723
Missing	851 (56.54)	489 (47.75)		251 (45.97)		97 (68.31)	
Physician global disease activity (0–10) (mean (SD))	4.88 (2.14)	4.87 (2.14)	0.926	4.87 (1.99)	0.962	5.02 (2.13)	0.547
Missing (%)	534 (35.48)	339 (33.11)		178 (32.6)		55 (38.73)	
Number of tender joints 28 (0–28) (mean (SD))	6.31 (6.32)	7.20 (6.97)	0.001	7.19 (6.7)	0.007	5.69 (6.15)	0.260
Missing (%)	10 (0.66)	6 (0.59)		3 (0.55)		2 (1.41)	
Number of swollen joints 28 (0–28) (mean (SD))	6.68 (5.9)	6.71 (5.63)	0.893	6.30 (5.50)	0.198	7.45 (6.66)	0.139
Missing (%)	7 (0.47)	2 (0.2)		2 (0.37)		1 (0.7)	
DAS28-ESR (mean (SD))	4.23 (1.42)	4.39 (1.41)	0.007	4.41 (1.35)	0.011	4.22 (1.57)	0.946
Missing	81 (5.38)	43 (4.2)		29 (5.31)		6 (4.23)	
DAS28-CRP (mean (SD))	3.92 (1.20)	4.07 (1.21)	0.035	4.12 (1.12)	0.016	3.90 (1.13)	0.906
Missing	860 (57.14)	495 (48.34)		252 (46.15)		98 (69.01)	
HAQ (mean (SD))	0.96 (0.71)	1.07 (0.72)	<0.001	1.18 (0.75)	<0.001	1.06 (0.74)	0.125
Missing	122 (8.11)	104 (10.16)		71 (13)		11 (7.75)	
Euro-QoL (mean (SD))	65.85 (19.31)	60.75 (21.80)	<0.001	59.02 (22.79)	<0.001	64.74 (18.16)	0.735
Missing	945 (62.79)	587 (57.32)		308 (56.41)		105 (73.94)	
SF12-pcs (mean (SD))	36.47 (10.27)	34.87 (9.57)	<0.001	34.16 (9.79)	<0.001	34.78 (10.62)	0.088
Missing	262 (17.41)	208 (20.31)		131 (23.99)		24 (16.9)	
SF12-mcs (mean (SD))	46.01 (11.56)	45.31 (12.01)	0.185	44.80 (12.33)	0.070	45.34 (12.28)	0.552
Missing	262 (17.41)	208 (20.31)		131 (23.99)		24 (16.9)	

Values are the number and column percentages, unless otherwise specified. Significance tests compared the underweight, overweight, and obese categories to the normal weight group using chi-squared test for categorical variables and *t*-test for continuous variables, except for the Wilcoxon test for ESR and CRP. For the tests, the missing values did not function as a grouping variable. Abbreviations: *n*—sample size; SD—standard deviation; IQR—interquartile range; RF+—rheumatoid factor positive; anti-CCP+—anti-cyclic citrullinated peptide positive; ESR—erythrocyte sedimentation rate; CRP—C-reactive protein; DAS28—28-joint Disease Activity Score; HAQ—Health Assessment Questionnaire; Euro-QoL—European Quality of Life instrument; SF12—Short-Form 12 health survey; pcs—physical component summary; mcs—mental component summary.

## Data Availability

Restrictions apply to the availability of these data. Data were obtained from the Swiss Clinical Quality Management in Rheumatic Diseases (SCQM) and are available after having received approval and permission from the license holder (SCQM).

## Supplementary Materials

The following are available online at https://www.mdpi.com/article/10.3390/jcm10143194/s1: Supplementary Equations (S1)–(S6); Supplementary Figure S1. Flow chart reflecting the patient inclusion process; Supplementary Table S1. Characteristics of psoriatic arthritis (PsA) patients starting their first biologic or targeted synthetic disease-modifying anti-rheumatic drug (b/tsDMARD) treatment. Additional variables to complement Table 2 and Table 3; Supplementary Table S2. Characteristics of rheumatoid arthritis (RA) patients starting their first biologic or targeted synthetic disease-modifying anti-rheumatic drug (b/tsDMARD) treatment. Additional variables to complement Table 5 and Table 6; Supplementary Table S3. First and second biologic or targeted synthetic disease-modifying anti-rheumatic drug (b/tsDMARD) treatment among the psoriatic arthritis (PsA) patients. Only those PsA patients with recorded information on both treatments were included; Supplementary Table S4. First and second biologic or targeted synthetic disease-modifying anti-rheumatic drug (b/tsDMARD) treatment among the rheumatoid arthritis (RA) patients. Only those RA patients with recorded information on both treatments were included.
