# Supplementary material for: Comparison of Psoriatic Arthritis and Rheumatoid Arthritis Patients across Body Mass Index Categories in Switzerland
Source: J Clin Med. 2021 Jul 20;10(14):3194. doi: 10.3390/jcm10143194 (PMC8304983; doi:10.3390/jcm10143194)
Supplement: Supplementary file 1 [file jcm-10-03194-s001.zip › jcm-1240910-supplementary.pdf]

Journal of
Clinical Medicine

MDPI

Supplementary Material

Comparison of Psoriatic Arthritis and Rheumatoid Arthritis Patients across Body Mass Index Categories in Switzerland

Enriqueta Vallejo-Yagüe [1], Theresa Burkard [1], Burkhard Möller [2], Axel Finckh [3] and Andrea M. Burden [1,*]

[1] Department of Chemistry and Applied Biosciences, Institute of Pharmaceutical Sciences, ETH Zurich, CH-8093 Zurich, Switzerland; enriqueta.vallejo@pharma.ethz.ch (E.V.-Y.); theresa.burkard@pharma.ethz.ch (T.B.)
[2] Rheumatology, Immunology and Allergy, Inselspital, University Hospital of Bern, CH-3010 Bern, Switzerland; burkhard.moeller@insel.ch
[3] Division of Rheumatology, University Hospitals of Geneva, CH-1206 Geneva, Switzerland; axel.finckh@hcuge.ch
* Correspondence: Correspondence: andrea.burden@pharma.ethz.ch; Tel.: +41-76-685-22-56

Supplementary Equations

$$\boldsymbol{BMI = \frac{weight\ Kg}{height\ m^2}} \quad \text{(S1)}$$

$$DAS28esr = (0.56 \times \sqrt{tjc28} + 0.28 \times \sqrt{sjc28} + 0.7 \times ln(esr)) \times 1.08 + 0.16 \quad \text{(S2)}$$

$$DAS28crp = \left(0.56 \times \sqrt{tjc28} + 0.28 \times \sqrt{sjc28} + 0.36 \times ln(crp + 1)\right) \times 1.10 + 1.15 \quad \text{(S3)}$$

$$DAPSA = sjc66 + tjc68 + PatActivity + PatPain + crp \quad \text{(S4)}$$

$$cDAPSA = sjc66 + tjc68 + PatActivity + PatPain \quad \text{(S5)}$$

MDA is achieved if at least 5 of the following items score positive: PatActivity (<2); PatPain (<1.5); HAQ (≤0.5); sjc (≤1); tjc (≤1); enthenesis (≤1); Skin manifestation none or almost none. (S6)

Abbreviations used in the above equations: BMI body mass index; DAS28 Disease Activity Score 28; DAPSA Disease Activity in Psoriasis Arthritis score; cDAPSA clinical DAPSA; MDA Minimal Disease Activity; swc28 Number of swollen joints, counting 28; swc66 Number of swollen joints, counting 66; tjc28 Number of tender joints, counting 28; tjc68 Number of tender joints, counting 68; esr Erythrocyte sedimentation rate; crp C-reactive protein (mg/dl); PatActivity Patient's assessment of disease activity on average the last 24 h (0 very well - 10 very poor); PatPain Patient's assessment of joint pain on average the last 24 h (0 very well - 10 very poor).

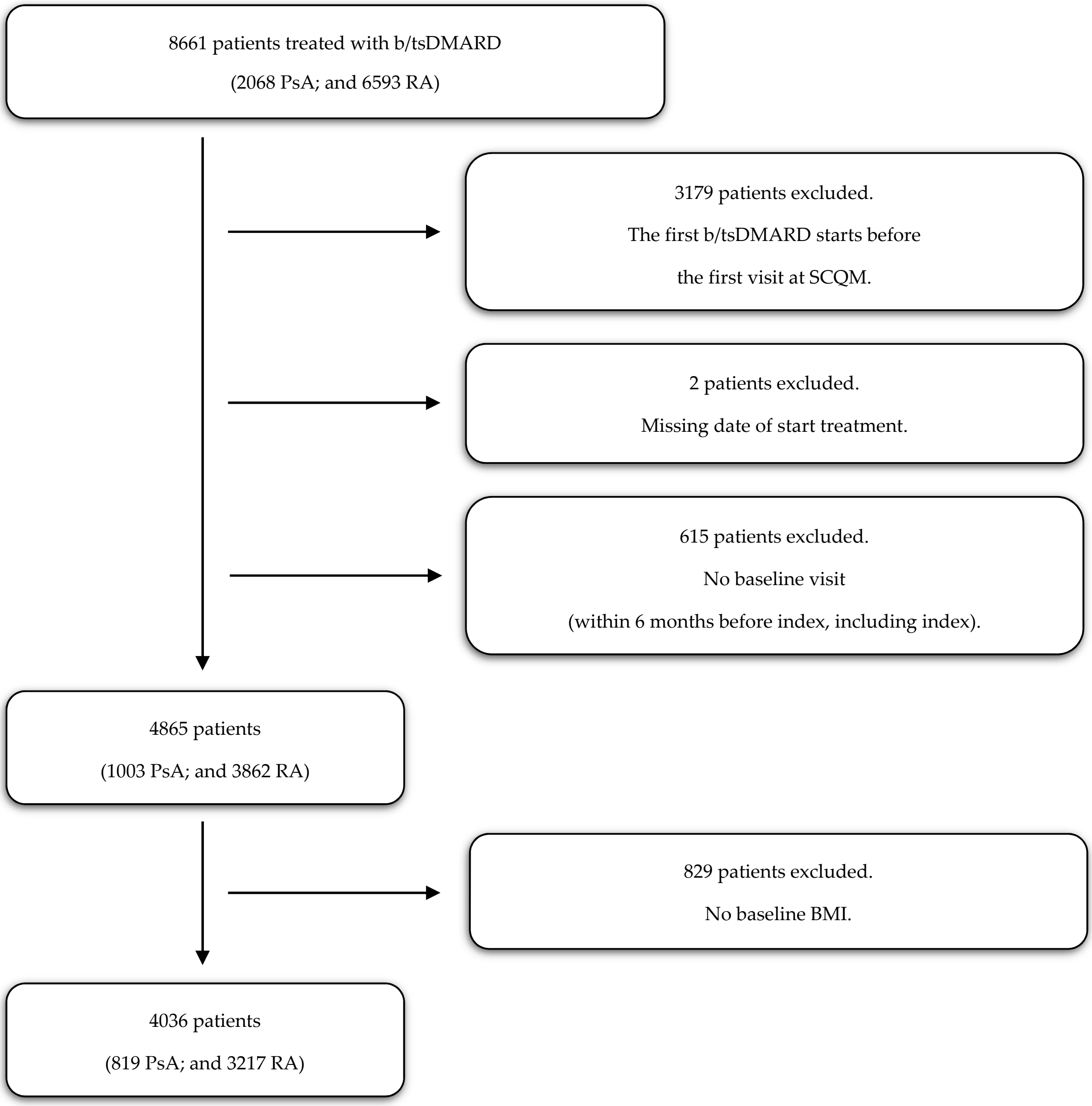

Supplementary Figure S1. Flow chart reflecting the patient inclusion process. Abbreviations: PsA psoriatic arthritis; RA rheumatoid arthritis; b/tsDMARD biologic or targeted synthetic disease modifying anti-rheumatic drug; SCQM Swiss Clinical Quality Management in Rheumatic Diseases; BMI body mass index.

Supplementary Table S1. Characteristics of psoriatic arthritis (PsA) patients starting their first biologic or targeted synthetic disease-modifying anti-rheumatic drug (b/tsDMARD) treatment. Additional variables to complement Table 2 and Table 3.

	Normal Weight (*n* = 325)	Overweight (*n* = 299)	*p*	Obese (*n* = 195)	*p*
Family history	138 (42.46)	114 (38.13)	0.307	73 (37.44)	0.299
Smoker	82 (25.23)	92 (30.77)	0.292	54 (27.69)	0.911
missing	175 (53.85)	149 (49.83)		99 (50.77)	
Alcohol	12 (3.69)	20 (6.69)	0.173	8 (4.10)	1.000
missing	166 (51.08)	143 (47.83)		96 (49.23)	
Schooling			0.001		0.006
compulsory school	49 (15.08)	64 (21.4)		43 (22.05)	
vocational training	134 (41.23)	137 (45.82)		81 (41.54)	
university studies	83 (25.54)	43 (14.38)		28 (14.36)	
missing	59 (18.15)	55 (18.39)		43 (22.05)	
Daily time walking/bicycling/similar activities outdoors			0.143		0.007
none	5 (1.54)	10 (3.34)		11 (5.64)	
<30min	40 (12.31)	53 (17.73)		36 (18.46)	
30to60min	72 (22.15)	57 (19.06)		32 (16.41)	
>60min	41 (12.62)	37 (12.37)		20 (10.26)	
missing	167 (51.38)	142 (47.49)		96 (49.23)	
Power sports			0.019		<0.001
none	45 (13.85)	70 (23.41)		56 (28.72)	
<1h	32 (9.85)	26 (8.7)		18 (9.23)	
1to2h	53 (16.31)	35 (11.71)		18 (9.23)	
>2h	30 (9.23)	26 (8.7)		6 (3.08)	
missing	165 (50.77)	142 (47.49)		97 (49.74)	
Index year (mean (SD))	2011.46 (4.31)	2011.69 (3.99)	0.478	2012.12 (4.20)	0.087
Reason for 1st b/tsDMARD stop			0.382		0.024
adverse event	29 (8.92)	31 (10.37)		25 (12.82)	
not effective	72 (22.15)	83 (27.76)		46 (23.59)	
remission	16 (4.92)	16 (5.35)		1 (0.51)	
other/missing	58 (17.85)	43 (14.38)		31 (15.9)	
not info on stop date	150 (46.15)	126 (42.14)		92 (47.18)	

Values are the number and column percentage, unless otherwise specified. Significance tests compared the overweight and obese categories to the normal weight group using chi-squared test for categorical variables, and t-test for continuous variables. For the tests, missing values did not function as a grouping variable. Abbreviations: n—sample size; SD—Standard deviation.

Supplementary Table S2. Characteristics of rheumatoid arthritis (RA) patients starting their first biologic or targeted synthetic disease-modifying anti-rheumatic drug (b/tsDMARD) treatment. Additional variables to complement Table 5 and Table 6.

	**Normal Weight (*n* = 1505)**	**Overweight (*n* = 1024)**	***p***	**Obese (*n* = 546)**	***p***	**Underweight (*n* = 142)**	***p***
Family history	337 (22.39)	228 (22.27)	0.979	110 (20.15)	0.304	37 (26.06)	0.373
Smoker	412 (27.38)	328 (32.03)	0.260	170 (31.14)	0.280	27 (19.01)	0.569
missing	822 (54.62)	509 (49.71)		282 (51.65)		93 (65.49)	
Alcohol	87 (5.78)	67 (6.54)	0.660	23 (4.21)	0.252	7 (4.93)	0.892
missing	495 (32.89)	307 (29.98)		192 (35.16)		50 (35.21)	
Schooling			<0.001		<0.001		0.022
compulsory school	228 (15.15)	237 (23.14)		139 (25.46)		10 (7.04)	
vocational training	612 (40.66)	388 (37.89)		183 (33.52)		62 (43.66)	
university studies	170 (11.3)	85 (8.3)		31 (5.68)		21 (14.79)	
missing	495 (32.89)	314 (30.66)		193 (35.35)		49 (34.51)	
Daily time walking/bicycling/ similar activities outdoors			0.495		0.037		0.077
none	127 (8.44)	91 (8.89)		63 (11.54)		17 (11.97)	
<30min	295 (19.6)	210 (20.51)		97 (17.77)		17 (11.97)	
30to60min	388 (25.78)	295 (28.81)		143 (26.19)		42 (29.58)	
>60min	247 (16.41)	154 (15.04)		69 (12.64)		21 (14.79)	
missing	448 (29.77)	274 (26.76)		174 (31.87)		45 (31.69)	
Power sports			0.073		0.007		0.436
none	501 (33.29)	393 (38.38)		201 (36.81)		53 (37.32)	
<1h	174 (11.56)	122 (11.91)		65 (11.9)		14 (9.86)	
1to2h	216 (14.35)	141 (13.77)		71 (13)		15 (10.56)	
>2h	159 (10.56)	86 (8.4)		31 (5.68)		12 (8.45)	
missing	455 (30.23)	282 (27.54)		178 (32.6)		48 (33.8)	
Index year (mean (SD))	2007.75 (5.09)	2008.52 (5.04)	<0.001	2009.05 (4.86)	<0.001	2006.36 (5.19)	0.002
Reason for 1st b/tsDMARD stop			0.111		0.007		0.562
adverse event	148 (9.83)	103 (10.06)		61 (11.17)		9 (6.34)	
not effective	297 (19.73)	214 (20.9)		125 (22.89)		25 (17.61)	
remission	57 (3.79)	45 (4.39)		28 (5.13)		7 (4.93)	
other/missing	354 (23.52)	195 (19.04)		93 (17.03)		26 (18.31)	
not info on stop date	649 (43.12)	467 (45.61)		239 (43.77)		75 (52.82)	

Values are the number and column percentage, unless otherwise specified. Significance tests compared the underweight, overweight and obese categories to the normal weight group using chi-squared test for categorical variables, and t-test for continuous variables. For the tests, missing values did not function as a grouping variable. Abbreviations: n—sample size; SD—Standard deviation.

Supplementary Table S3. First and second biologic or targeted synthetic disease-modifying anti-rheumatic drug (b/tsDMARD) treatment among the psoriatic arthritis (PsA) patients. Only those PsA patients with recorded information on both treatments were included.

1st biologic/tsDMARD	2nd biologic/tsDMARD	Normal Weight (*n* = 155)	Overweight (*n* = 140)	Obese (*n* = 90)
**adalimumab (*n* (%))**		**54 (34.84)**	**62 (44.29)**	**34 (37.78)**
adalimumab	adalimumab	12 (22.22)	13 (20.97)	5 (14.71)
adalimumab	etanercept	14 (25.93)	17 (27.42)	11 (32.35)
adalimumab	infliximab	7 (12.96)	7 (11.29)	3 (8.82)
adalimumab	certolizumab	-	5 (8.06)	2 (5.88)
adalimumab	golimumab	10 (18.52)	12 (19.35)	9 (26.47)
adalimumab	abatacept	1 (1.85)	-	-
adalimumab	secukinumab	4 (7.41)	1 (1.61)	1 (2.94)
adalimumab	ixekizumab	1 (1.85)	1 (1.61)	-
adalimumab	ustekinumab	4 (7.41)	-	2 (5.88)
adalimumab	apremilast	1 (1.85)	6 (9.68)	1 (2.94)
**etanercept (*n* (%))**		**56 (36.13)**	**29 (20.71)**	**30 (33.33)**
etanercept	adalimumab	27 (48.21)	10 (34.48)	14 (46.67)
etanercept	etanercept	10 (17.86)	6 (20.69)	3 (10.00)
etanercept	infliximab	8 (14.29)	1 (3.45)	1 (3.33)
etanercept	certolizumab	2 (3.57)	-	-
etanercept	golimumab	6 (10.71)	7 (24.14)	9 (30.00)
etanercept	tocilizumab	-	1 (3.45)	-
etanercept	secukinumab	-	2 (6.90)	-
etanercept	ustekinumab	-	1 (3.45)	3 (10.00)
etanercept	apremilast	3 (5.36)	1 (3.45)	-
**infliximab (*n* (%))**		**16 (10.32)**	**20 (14.29)**	**7 (7.78)**
infliximab	adalimumab	6 (37.50)	6 (30)	2 (28.57)
infliximab	etanercept	3 (18.75)	4 (20)	-
infliximab	infliximab	1 (6.25)	4 (20)	3 (42.86)
infliximab	golimumab	5 (31.25)	4 (20)	2 (28.57)
infliximab	abatacept	-	1 (5)	-
infliximab	ustekinumab	1 (6.25)	1 (5)	-
**certolizumab (*n* (%))**		**3 (1.94)**	**2 (1.43)**	**4 (4.44)**
certolizumab	etanercept	1 (33.33)	2 (100)	1 (25.00)
certolizumab	golimumab	1 (33.33)	-	2 (50.00)
certolizumab	ustekinumab	-	-	1 (25.00)
certolizumab	apremilast	1 (33.33)	-	-
**golimumab (*n* (%))**		**18 (11.61)**	**20 (14.29)**	**6 (6.67)**
golimumab	adalimumab	3 (16.67)	6 (30)	2 (33.33)
golimumab	etanercept	2 (11.11)	3 (15)	2 (33.33)
golimumab	infliximab	1 (5.56)	2 (10)	-
golimumab	certolizumab	2 (11.11)	1 (5)	-
golimumab	golimumab	3 (16.67)	4 (20)	1 (16.67)
golimumab	secukinumab	-	1 (5)	-
golimumab	ixekizumab	1 (5.56)	-	-
golimumab	ustekinumab	5 (27.78)	-	-
golimumab	apremilast	1 (5.56)	3 (15)	1 (16.67)
**abatacept (*n* (%))**		**1 (0.65)**	**1 (0.71)**	**0 (0)**
abatacept	infliximab	1 (100.00)	-	-
abatacept	tocilizumab	-	1 (100)	-

**tocilizumab (*n* (%))**		**-**	**1 (0.71)**	**-**
tocilizumab	infliximab	-	1 (100)	-
**secukinumab (*n* (%))**		**1 (0.65)**	**1 (0.71)**	**2 (2.22)**
secukinumab	infliximab	1 (100)	-	-
secukinumab	golimumab	-	-	1 (50)
secukinumab	ixekizumab	-	1 (100)	-
secukinumab	tofacitinib	-	-	1 (50)
**ustekinumab (*n* (%))**		**1 (0.65)**	**1 (0.71)**	**0 (0)**
ustekinumab	adalimumab	1 (100)	-	-
ustekinumab	etanercept	-	1 (100)	-
**apremilast (*n* (%))**		**5 (3.23)**	**3 (2.14)**	**7 (7.78)**
apremilast	adalimumab	3 (60)	-	3 (42.86)
apremilast	etanercept	-	-	2 (28.57)
apremilast	golimumab	-	-	1 (14.29)
apremilast	secukinumab	1 (20)	2 (66.67)	-
apremilast	ustekinumab	-	1 (33.33)	-
apremilast	apremilast	1 (20)	-	1 (14.29)

Supplementary Table S4. First and second biologic or targeted synthetic disease-modifying anti-rheumatic drug (b/tsDMARD) treatment among the rheumatoid arthritis (RA) patients. Only those RA patients with recorded information on both treatments were included.

1st biologic/tsDMARD	2nd biologic/tsDMARD	Normal weight (*n* = 749)	Overweight (*n* = 479)	Obese (*n* = 259)	Underweight (*n* = 59)
**adalimumab (*n* (%))**		**228 (30.44)**	**156 (32.57)**	**82 (31.66)**	**16 (27.12)**
adalimumab	adalimumab	32 (14.04)	20 (12.82)	8 (9.76)	4 (25.00)
adalimumab	etanercept	63 (27.63)	43 (27.56)	23 (28.05)	6 (37.50)
adalimumab	infliximab	19 (8.33)	10 (6.41)	4 (4.88)	-
adalimumab	certolizumab	5 (2.19)	2 (1.28)	3 (3.66)	-
adalimumab	golimumab	13 (5.70)	13 (8.33)	5 (6.10)	4 (25.00)
adalimumab	rituximab	42 (18.42)	19 (12.18)	15 (18.29)	1 (6.25)
adalimumab	abatacept	29 (12.72)	17 (10.90)	13 (15.85)	-
adalimumab	tocilizumab	17 (7.46)	26 (16.67)	5 (6.10)	-
adalimumab	apremilast	1 (0.44)	-	-	-
adalimumab	baricitinib	-	1 (0.64)	-	1 (6.25)
adalimumab	tofacitinib	7 (3.07)	5 (3.21)	6 (7.32)	-
**etanercept (*n* (%))**		**266 (35.51)**	**156 (32.57)**	**72 (27.8)**	**22 (37.29)**
etanercept	adalimumab	102 (38.35)	58 (37.18)	30 (41.67)	11 (50.00)
etanercept	etanercept	34 (12.78)	14 (8.97)	5 (6.94)	1 (4.55)
etanercept	infliximab	32 (12.03)	18 (11.54)	8 (11.11)	4 (18.18)
etanercept	certolizumab	5 (1.88)	3 (1.92)	1 (1.39)	-
etanercept	golimumab	11 (4.14)	10 (6.41)	7 (9.72)	-
etanercept	rituximab	33 (12.41)	17 (10.90)	10 (13.89)	1 (4.55)
etanercept	abatacept	16 (6.02)	9 (5.77)	4 (5.56)	2 (9.09)
etanercept	anakinra	-	1 (0.64)	-	-
etanercept	tocilizumab	19 (7.14)	15 (9.62)	1 (1.39)	3 (13.64)
etanercept	baricitinib	2 (0.75)	-	1 (1.39)	-
etanercept	tofacitinib	12 (4.51)	11 (7.05)	5 (6.94)	-
**infliximab (*n* (%))**		**139 (18.56)**	**87 (18.16)**	**40 (15.44)**	**16 (27.12)**
infliximab	adalimumab	45 (32.37)	28 (32.18)	16 (40)	9 (56.25)
infliximab	etanercept	29 (20.86)	17 (19.54)	4 (10)	3 (18.75)
infliximab	infliximab	16 (11.51)	9 (10.34)	-	1 (6.25)
infliximab	certolizumab	1 (0.72)	-	-	-
infliximab	golimumab	4 (2.88)	5 (5.75)	1 (2.5)	-
infliximab	rituximab	15 (10.79)	14 (16.09)	10 (25)	1 (6.25)
infliximab	abatacept	15 (10.79)	5 (5.75)	5 (12.5)	-
infliximab	tocilizumab	13 (9.35)	9 (10.34)	4 (10)	2 (12.5)
infliximab	tofacitinib	1 (0.72)	-	-	-
**certolizumab (*n* (%))**		**16 (2.14)**	**6 (1.25)**	**7 (2.7)**	**0 (0)**
certolizumab	adalimumab	1 (6.25)	-	-	-
certolizumab	etanercept	3 (18.75)	2 (33.33)	1 (14.29)	-
certolizumab	infliximab	2 (12.50)	-	-	-
certolizumab	certolizumab	2 (12.50)	1 (16.67)	1 (14.29)	-
certolizumab	golimumab	-	-	1 (14.29)	-
certolizumab	rituximab	1 (6.25)	-	-	-
certolizumab	abatacept	2 (12.50)	1 (16.67)	2 (28.57)	-
certolizumab	tocilizumab	2 (12.50)	1 (16.67)	1 (14.29)	-
certolizumab	ixekizumab	-	-	1 (14.29)	-
certolizumab	baricitinib	1 (6.25)	-	-	-
certolizumab	tofacitinib	2 (12.5)	1 (16.67)	-	-

**golimumab (*n* (%))**		**40 (5.34)**	**22 (4.59)**	**23 (8.88)**	**3 (5.08)**
golimumab	adalimumab	6 (15)	1 (4.55)	2 (8.70)	-
golimumab	etanercept	8 (20)	4 (18.18)	4 (17.39)	1 (33.33)
golimumab	infliximab	2 (5)	2 (9.09)	2 (8.70)	-
golimumab	certolizumab	-	2 (9.09)	1 (4.35)	-
golimumab	golimumab	7 (17.5)	2 (9.09)	3 (13.04)	1 (33.33)
golimumab	rituximab	1 (2.5)	2 (9.09)	2 (8.70)	-
golimumab	abatacept	8 (20)	3 (13.64)	3 (13.04)	-
golimumab	tocilizumab	4 (10)	6 (27.27)	3 (13.04)	1 (33.33)
golimumab	tofacitinib	4 (10)	-	3 (13.04)	-
**rituximab (*n* (%))**		**11 (1.47)**	**7 (1.46)**	**4 (1.54)**	**0 (0)**
rituximab	adalimumab	2 (18.18)	1 (14.29)	-	-
rituximab	etanercept	1 (9.09)	1 (14.29)	-	-
rituximab	infliximab	-	1 (14.29)	-	-
rituximab	certolizumab	1 (9.09)	-	-	-
rituximab	rituximab	4 (36.36)	3 (42.86)	1 (25)	-
rituximab	abatacept	-	-	2 (50)	-
rituximab	tocilizumab	2 (18.18)	-	-	-
rituximab	baricitinib	-	1 (14.29)	-	-
rituximab	tofacitinib	1 (9.09)	-	1 (25)	-
**abatacept (*n* (%))**		**23 (3.07)**	**21 (4.38)**	**19 (7.34)**	**1 (1.69)**
abatacept	adalimumab	1 (4.35)	-	-	-
abatacept	etanercept	2 (8.70)	5 (23.81)	1 (5.26)	-
abatacept	infliximab	1 (4.35)	-	1 (5.26)	-
abatacept	golimumab	-	2 (9.52)	2 (10.53)	1 (100)
abatacept	rituximab	4 (17.39)	4 (19.05)	2 (10.53)	-
abatacept	abatacept	7 (30.43)	2 (9.52)	4 (21.05)	-
abatacept	tocilizumab	6 (26.09)	7 (33.33)	6 (31.58)	-
abatacept	baricitinib	-	1 (4.76)	1 (5.26)	-
abatacept	tofacitinib	2 (8.70)	-	2 (10.53)	-
**tocilizumab (*n* (%))**		**19 (2.54)**	**17 (3.55)**	**7 (2.70)**	**1 (1.69)**
tocilizumab	adalimumab	2 (10.53)	1 (5.88)	2 (28.57)	-
tocilizumab	etanercept	1 (5.26)	2 (11.76)	-	-
tocilizumab	certolizumab	1 (5.26)	-	-	-
tocilizumab	golimumab	-	3 (17.65)	-	-
tocilizumab	rituximab	3 (15.79)	-	2 (28.57)	-
tocilizumab	abatacept	5 (26.32)	2 (11.76)	2 (28.57)	1 (100)
tocilizumab	tocilizumab	4 (21.05)	4 (23.53)	1 (14.29)	-
tocilizumab	baricitinib	-	1 (5.88)	-	-
tocilizumab	tofacitinib	3 (15.79)	4 (23.53)	-	-
**tofacitinib (*n* (%))**		**7 (0.93)**	**7 (1.46)**	**5 (1.93)**	**0 (0)**
tofacitinib	adalimumab	1 (14.29)	-	1 (20)	-
tofacitinib	certolizumab	-	1 (14.29)	-	-
tofacitinib	golimumab	2 (28.57)	1 (14.29)	2 (40)	-
tofacitinib	rituximab	1 (14.29)	-	-	-
tofacitinib	abatacept	1 (14.29)	1 (14.29)	-	-
tofacitinib	tocilizumab	1 (14.29)	3 (42.86)	-	-
tofacitinib	baricitinib	1 (14.29)	-	1 (20)	-
tofacitinib	tofacitinib	-	1 (14.29)	1 (20)	-

Copyright: © 2021 by the authors. Licensee MDPI, Basel, Switzerland. This article is an open access article distributed under the terms and conditions of the Creative Commons Attribution (CC BY) license (http://creativecommons.org/licenses/by/4.0/).
